# The special issue on cancer and evolution: Lessons learned

**DOI:** 10.1111/eva.13040

**Published:** 2020-07-13

**Authors:** James DeGregori

**Affiliations:** ^1^ Department of Biochemistry and Molecular Genetics Department of Immunology and Microbiology University of Colorado Anschutz Medical Campus Aurora CO USA

**Keywords:** cancer evolution, cancer risk and oncogenesis, somatic evolution, transmissible cancers

## Abstract

This special issue of evolutionary applications focused on the evolution of cancer has provided a wealth of different viewpoints and results from leaders in the field. Together, these papers emphasize the importance of a broad perspective in order to understand why we and other animals get cancer, how it evolves within an individual, and what we can do about it. We can no longer take reductionist approaches that consider only the cancer cells and their genes. Instead, we need to understand how millions of years of evolution have guided strategies that shape cancer risk, why cancer risk varies across different animals, how cancer risk can vary in a population and be influenced by ecology (and influence this ecology), and of course how cancers evolve within us and the evolutionarily informed strategies to counter their impact. My goal here will be to “bring it all home,” providing a refresher of lessons learned with added kibitzing.

## 1 EVOLUTIONARY UNDERSTANDING OF CANCER RISK

Evolution has shaped stem cell pool organizations and dynamics both to optimize tissue function and to prevent the development of malignancies that could impair organismal fitness. Birtwell et al. (This volume Birtwell et al., n.d.) highlight the critical need to determine the distribution of fitness effects of mutations in epithelial stem cells (modeled here for intestinal crypts) in order to understand how competition within a crypt or between crypts dictates cancer risk. They used an agent‐based microsimulation model to demonstrate that competition between crypts (for small stem cell pool sizes) and within crypts (for larger pool sizes) suppresses mutator clones when most non‐neutral mutations are deleterious (as they likely are). The higher the proportion of deleterious mutations, the lower the odds of tumor suppressor gene inactivation, coinciding with reduced emergence of mutator clones. This study can help explain how stem cell pool organization (neither too big nor too small), and competition within and between the units, can minimize carcinogenesis. Can their model be used to explain a quandary arising from recent studies of clonal prevalence in normal tissues? While around 1% of colonic crypts are fixed for oncogenic mutations (translating into ~ 100,000 oncogenically activated crypts in an adult) (Lee‐Six et al., 2019), this number is much higher for the esophagus and the endometrium in older adults (Martincorena et al., 2018; Moore et al., 2020; Yokoyama et al., 2019), suggesting stronger selection against such clones in the colon.

How does evolution accommodate different life history strategies, including body size and longevity, while mitigating associated risks like somatic decline and cancer? Erten and Kokko (This volume Erten and Kokko, n.d.) explore “ontogenetic management strategies” for somatic cells, and how these strategies differentially evolve in organisms dependent on body size. They model a range of strategies for in silico organisms, varying parameters of somatic cell strategies, adult body size, and extrinsic hazards. Somatic cell strategies include the Hayflick limit, the number of differentiation steps in cell lineage, the probability of asymmetric cell division, the probability that daughter cells differentiate, and DNA damage response threshold (and the odds of consequent cell death). There appear to be many ways to create a soma of different sizes, such as by varying the number of steps to full differentiation. Like real ones, in silico “animals” can die from various causes, including extrinsic factors, somatic maintenance failure, and, of course, cancer. They employ a genetic algorithm to determine optimal sets of strategies, with mutation of the strategies and evolution over generations. Different strategies can have tradeoffs, for example, those that enhance longevity, like telomere maintenance, may also increase cancer risk. Importantly, strategies that evolve in larger bodies function well in small bodied organisms, but not vice versus—those that evolve in small bodies tend to engender premature death in large bodied animals. It appears easier to downsize than to upsize. Somewhat mirroring the real world, optimized strategies tended to manage the soma well enough such that in silico creatures died of extrinsic causes before the intrinsic failures or cancer could bring about their end. For most humans, this calculus has changed, with aging‐associated intrinsic failures and cancer constituting the major causes of our demise. Sadly, in the era of COVID‐19, many countries are now experiencing more deaths from this extrinsic threat than other causes.

The renowned epidemiologist Sir Richard Peto famously noted that cancer risk does not scale with body size, despite expectations from the classical models of carcinogenesis (Nunney, (1999); Caulin & Maley, 2011). Using a multistage mathematical model, Nunney (This volume Nunney, n.d.) explores multiple different potential explanations for this paradox, including species‐specific differences in metabolic rate, mutation rates, immune surveillance, and the number of tumor suppressor barriers to transformation. They basically asked—what parameter changes would be required to bring the cancer rate of bigger animals (whale and human) down to that of a mouse? Their results suggest that size‐related changes in metabolic rates cannot explain the lack of cancer scaling with body size. Similarly, mutation rates would need to be reduced 3–5 orders of magnitude in larger animals to achieve similar cancer rates, at least under their assumptions. The immune system hypothesis would require an unrealistically high ability of larger animals to detect cancer cells, consistent with the relatively modest effects of clinical immune suppression on cancer incidence. In contrast, additional layers of tumor suppression, requiring additional steps in multistage carcinogenesis, can resolve Peto's Paradox and is consistent with results showing differences in the numbers of oncogenic events required to transform human and mouse cells (Rangarajan, Hong, Gifford, & Weinberg, 2004). The question remains—how can we explain differences in the numbers of required drivers for different cancers even within a species?

Indeed, there has been much debate in the cancer community concerning the basis of cancer incidence patterns: between different tissues, within a lifespan, and across the animal kingdom. Why do cancers that arise in various tissues with very different stem cell pools and, requiring highly variable numbers of oncogenic mutations, show such similar late‐life patterns of incidence in humans? And why are animal species with huge variance in body size and lifespans mostly able to delay cancer risk till what would be postreproductive ages in the “wild” (the Peto's Paradox described above)? Rozhok and DeGregori (This volume Rozhok and DeGregori, n.d.) propose that three orthogonal evolutionary processes controlling (a) somatic mutation occurrence, (b) species‐specific life history traits (strategies for tissue maintenance and tumor suppression that maximize the odds of reproductive success), and (c) rates of physiological aging determine cancer rates across species and within the lifetimes of individuals (tissue decline in old age promotes selection for adaptive and sometimes oncogenic mutations). They attempt to reconcile what appear to be divergent observations of mutation rates, cancer susceptibility across animals, and similar aging‐associated rates for many cancers with very different somatic evolutionary parameters into a unified framework.

Aging and other contexts (like due to cigarette smoking) both increase mutation prevalence (and thus heritable variability) and tissue environmental changes that contribute to dramatic increases in cancer risk (Laconi, Marongiu, & DeGregori, 2020). Gatenby and Brown (This volume Gatenby and Brown, n.d.) describe how normal cells can accumulate mutations, but that these mutations do not promote somatic evolution as the cells only possess the fitness function of the host animal, at least in young healthy tissues. In this manner, a soma serves its one true master—the germline, with the evolved objective of maximizing germline transmission. Only upon insult (inflammation, damage, aging, etc) is there a loss of this tissue control, and the cells (even if temporarily) acquire a self‐defined fitness function, akin to a speciation event. Thus, aging and other insults promote cancers by facilitating the transition from host‐defined to self‐defined fitness. They also provide fascinating insight into the myriad of sources of information content in a cell, beyond its DNA, such as transmembrane ion distributions. Their model adds to a growing appreciation that cancer evolution is about more than just mutations, but requires overcoming hurdles evolved by animals to maintain functional tissues despite mutation accumulation (DeGregori, 2011; Gatenby & Gillies, 2008).

As argued above, tissue microenvironments and how they change during life and following insults exert substantial influences on somatic evolution and cancer risk, notwithstanding the common adherence to a mutation‐centric explanation of cancer risk (e.g., Tomasetti, Li, & Vogelstein, (2017)). Solary and Lapane (This volume Solary and Lapane, n.d.) describe how clones often driven by putatively oncogenic mutations and, even genetically complex carcinomas, accumulate in our tissues (even dominating tissues) as we age. But what determines whether these clones contribute to cancer or, as is the case for the VAST majority of these clones, not? The authors describe how normal (youthful) tissue architecture can impair cancer development and the many tissue disturbances that can promote the malignant evolution of these clones. A normal tissue microenvironment not only restrains oncogenesis by suppressing *selection* for malignant phenotypes, but can even *normalize* cells with malignant genotypes (i.e., stifling the malignant phenotype that would otherwise result). Malignant clonal emergence often requires tissue disruptions that result from wounding, UV light, therapies, other extrinsic exposures (e.g., from smoking), obesity, and of course aging‐related tissue decline. Commonalities of these contexts include increased inflammation and reduced cell competition, and often stromal cell senescence and gut dysbiosis. Cancers evolve to manipulate their own microenvironment, altering stromal, immune, soluble and matrix components, and luckily the vast majority of oncogene‐driven clonal expansions fail to successfully do so. The ubiquitous presence of oncogenic mutations in our tissues raises important questions about the forces controlling cancer development and has additional implications toward early detection. For cancer prevention, while avoiding mutagenic exposures is of course still very much advisable, more efforts should be invested in developing interventions that alter tissue environments to be less cancer promoting. Strategies to target malignant cells need to be complemented by interventions that control the microenvironment and thus the direction of somatic evolution.

Racial disparities in cancer risk and outcomes are well described (O'Keefe, Meltzer, & Bethea, 2015). Understanding the basis of such disparities will be necessary to develop mitigations. By mining TCGA data (estimating ancestry from SNPs), Schenk et al. (This volume Schenk et al., n.d.) examined how genetic ancestry influences lung cancer pathogenesis and found that African Americans (AA) that develop lung adenocarcinomas (but not squamous cell carcinomas) are significantly younger and smoke less than European Americans (EA). Despite being younger and having smoked less (on average), AAs developed adenocarcinomas that exhibited more nonsilent mutations, exhibited an increase in the CS4 mutation signature associated with cigarette smoking, and displayed more mutations in known cancer genes, than those in EAs. Effectively, each pack of cigarettes smoked appears to result in more mutations and earlier cancer development in AAs relative to EAs. This study shows that germline ancestry can impact mutational processes and likely somatic selection landscapes, although it is mysterious why such effects are not evident for lung squamous cell carcinomas. What is clear is that our evolutionary past has substantial influences on somatic evolutionary processes in our bodies.

## 2 AN EVOLUTIONARY TANGO—THE DYNAMICS OF CANCER CELLS WITHIN US AND WITH OTHER ENTITIES

The advent of single‐cell genomic technologies, including single cell RNAseq and DNAseq, has identified marked cellular heterogeneity with cancers, both phenotypic and genotypic (Lipinski et al., 2016; Marusyk, Janiszewska, & Polyak, 2020). This heterogeneity is driven by variable selective forces throughout the malignancy, influenced by microenvironmental variables including pH, oxygen, nutrients, immune cells, and other stromal cells, and even competition with other cancer cells. Notably, drift also plays a significant role, particularly at later stages of cancer evolution (Sun, Hu, & Curtis, 2018; Williams et al., 2018). Robert Noble et al. (This volume Noble et al., n.d.) leveraged computational modeling to better understand the conditions that dictate when cellular heterogeneity predicts future cancer growth. As they review, higher clonal diversity sometimes predicts poor outcomes for patients, likely due to the increased adaptability provided by diverse phenotypic variants in the face of challenges, including from therapies. But then sometimes it does not, perhaps due to selective sweeps by highly malignant clones or the general unpredictability of cancers. Their spatial, stochastic model follows tumor evolution in a 2D grid. They show that clonal diversity early in cancer evolution predicts higher growth rates later, while the opposite is true for diversity late in cancer evolution, perhaps due to clonal interference and the lack of selective sweeps. It is important to measure diversity across the tumor, not only at the edge, and to consider the mutation rate of the tumor, consistent with analyses of kidney cancers (Turajlic et al., 2018). To the extent that computational modeling can predict real cancer evolution, these results suggest that leveraging tumor heterogeneity for prognosis will require careful consideration of when and where this diversity is measured.

Clusters of metastasizing cells have been observed in humans and animal models, and thought to increase metastatic cell survival and seeding of distant sites (Cheung & Ewald, 2016). Campenni et al. (This volume Campenni et al., n.d.) use mechanistic agent‐based modeling to explore vulnerabilities of circulating tumor cell clusters, which contribute to metastases. The resiliency of clusters, including in response to microenvironmental threats including drugs, is positively associated with their density and size. These modeling studies should spur experimental approaches to test these associations, with the goal of discovering means to disrupt this group protection mechanism.

By some estimates, at least 15% (and likely more) of cancers have infectious origins (Ewald & Swain Ewald, 2013). These include cancers that are directly caused by viruses (e.g., liver, head and neck, and almost all cervical carcinomas) and others that emanate from the inflammation that accompanies pathogen infection (e.g., *Helicobacter pylori* and stomach cancers) (Fernandes et al., 2015; Lin, King, & Chung, 2015; Wu et al., 2010). While most people with these pathogens do not develop cancers, these infections are associated with a substantial increase in risk. But are microbes always the bad guys when it comes to cancer? Swain Ewald and Ewald (This volume Swain Ewald and Ewald, n.d.) outline their barrier theory of cancer, which incorporates organismal intrinsic mechanisms of tumor suppression (cell cycle arrest, apoptosis, regulation of telomerase, cell adhesion, and asymmetric cell division) with extrinsic factors (environment, including pathogens) which can alter these intrinsic defenses. Pathogens have indirectly evolved to abrogate cancer defense mechanisms, as these host mechanisms can often serve to limit pathogen persistence. As has become increasingly clear in recent years, microbes can be our friends. The authors revise their barrier theory to incorporate how a healthy microbiome can protect against cancers, adding to the list of their essential contributions to our well‐being (synthesis of certain vitamins, proper digestion, water absorption, barrier function, and immune regulation) (Rook & Dalgleish, 2011). An unhealthy gut microbiome can reduce barrier function and increase inflammation, which can promote cancer evolution. In contrast, a healthy microbiome produces short‐chain fatty acids like butyrate that promote barrier function and suppress inflammation. A Western diet high in red meat favors the unhealthy microbiota, while a high‐fiber diet provides fuel for butyrate‐producing bacteria. Mutualists can also antagonize cancer‐causing pathogens. In addition to evidence that your microbiota influences your cancer risk, the microbiota can influence immune function to substantially impact responses to anti‐cancer checkpoint therapies. However, we are far from understanding these connections, with different studies implicating different bacterial species as key determinants of cancer risk or immune responses. As always, the reality is more complex than what (necessarily) reductionist laboratory experiments suggest.

## 3 MEDICINE A LA DARWIN

An evolutionary understanding of cancer has the potential to transform how we prevent, manage, and treat this disease. A human cancer can be composed of on the order of a trillion cells, and thus virtually every possible mutation or gene loss/gain will be present within this population, particularly when one considers increases in mutation prevalence in cancer cells. Similarly, pathogens can also have high population sizes and high mutation rates. Thus, whether for cancer or a disease‐causing pathogen, resistance often develops in response to treatments designed to eradicate the problem, and evolutionary‐informed strategies are required to limit the development of therapy resistance (Gatenby and Brown, 2018). Merlo et al. (This volume Merlo et al., n.d.) evolved the yeast *Saccharomyces cerevisiae* under different selective pressures. They showed that simultaneously limiting three essential nutrients slows the rate of evolution on any one of the three selective pressures relative to each single selective pressure. These studies validate the concept of clonal interference, whereby multiple selective pressures lead to clonal competition that limits adaptation to any one pressure. In addition, they showed that strains evolved on a particular restriction, while more fit in the nutrient limited context, where less fit on regular media, consistent with a cost of resistance (a trade‐off). Genome sequencing revealed mechanisms underlying adaptation. Targeting multiple pathways in cancer cells or pathogens, assuming that a common mechanism of resistance is not likely, could substantially extend the period of treatment efficacy and survival (whether of a patient, a pet or a crop). So to get rid of your enemies, you have to get them to fight each other.

Most therapies for cancer seek the maximum tolerated dose (MTD), often established in the clinical trials that led to the approval of the drug regimen (Le Tourneau, Lee, & Siu, 2009). More recently, results from computational modeling, mouse models of cancer, and clinical trials have indicated that seeking the MTD may be misguided, not only due to excessive damage to normal tissues but also because such strategies will lead to fixation of therapy‐resistant cancer cells (West et al., 2020). While evolutionary‐informed strategies, such as adaptive therapy, can keep a cancer at bay for longer (and with less toxicity to the patient), the cost may be the abandonment of an attempt to cure (Hansen, Woods, & Read, 2017). So how to choose? Hansen and Read (This volume Hansen and Read, n.d.) explore this quandary. They model aggressive therapy with the intent to cure, but with the risk of earlier progression of drug resistant disease, versus containment strategy to manage resistance which uses competition to keep resistant cells at bay but where cure is unlikely. They show that this decision will depend on the probability of cure and the extent to which containment can delay resistant relapse, which are themselves dependent on mutation rate, cancer cell turnover kinetics, initial tumor burden, and the number of initial therapy‐resistant cells. These parameters are used to place patients on a cure‐progression plane; with clinical validation, and with the caveat that patients and their cancers are highly complex and thus difficult to parameterize, this tool could be useful for decision making. Finally, it would be interesting to add in additional components—how damage to the tissue microenvironment dependent on the intensity of therapy could engender selection for more aggressive cancer phenotypes and how differences in immune parameters can guide decisions.

Can cancer cells be coaxed into a state that is more sensitive to therapy—a sucker's gambit (Maley, Reid, & Forrest, 2004)? Girard et al. (This volume Girard et al., n.d.) explore a new strategy for directing the evolution of cancer cells through application of a DNA repair activator AsiDNA in a breast cancer cell line. AsiDNAs mimic double‐stranded DNA breaks, delivering a false signal that activates PARP and DNA PK pathways, leading to evolution of clones that downregulate these highly energy consuming pathways. Thus, evolvability appears to be reduced, and cancer cells actually evolve toward greater AsiDNA sensitivity, but without increases in somatic fitness parameters. While some of these changes are actualized in a xenograft model, it will critical to explore the effectiveness of this strategy in controlling tumor evolution and aggressiveness in vivo, perhaps in combination with other interventions.

Giant cells with grossly high DNA content have been observed in cancers for decades, and while shown to be stress resistant with increased pro‐metastatic phenotypes, their significance remains underappreciated. In fact, polyploidization is a strategy of stress resistance used across the tree of life (including in single‐celled eukaryotes) (Van de Peer, Mizrachi, & Marchal, 2017). Pienta et al. (This volume Pienta et al., n.d.) provide important new insight into poly‐aneuploid cancer cells (PACCs), which are understudied and certainly underappreciated, and their role in cancer adaptation, both as the evolving malignancy encounters host‐imposed hurdles and in response to therapies. The poly‐aneuploid state, and the resolution of this state through loss of genetic material or through evolution of redundant genes, provides increased heritable variability for adaptation to changing environments. Thus, PACCs could be key players in allowing both stress/therapy resistance and greater evolvability.

Kathleen Noble et al. (This volume Noble et al., n.d.) describe the utility of animal‐derived toxins as anti‐cancer therapeutics, leveraging the millions of years of natural selection that have honed these compounds for efficacy and stability. Thus, these compounds can have good pharmacodynamic and pharmacokinetic properties, two highly desired characteristics for a pharmacological agent. They describe anti‐cancer agents in development from insects, arachnids, amphibians, and marine organisms, diving into details for one compound each. These include melittin in bee venom (pro‐cell death and anti‐proliferative through multiple cellular targets), chlorotoxin from the Israeli Deathstalker Scorpion (which selectively binds cancer cells, facilitating cancer detection and targeting), Huchansu from the Chinese Bufo toad (a complex mixture of chemicals with a similarly complex mechanism of action), and trabectedin from the Mangrove Tunicate (which binds and distorts DNA, altering transcription factor binding). While the other toxins are still under investigation, given its efficacy trabectedin was approved by the FDA for treatment of liposarcoma and leiomyosarcoma. Challenges remain for most of these agents, including for efficacy and toxicity, as animals did not evolve to produce these compounds as anti‐cancer agents, but as toxins to ward off enemies. Can we learn to better harness these poisons for the benefit of patients?

## 4 VIVE LA DIFFERENCE (ET LES POINTS COMMUNS)

Cancer will impact the fitness of an organism in the wild well before it would cause death under protected conditions (Ujvari, Roche, & Fdr, 2017). While we often consider the impact that a cancer has on an individual, very little attention has been paid to cancer's effects on interspecies interactions. Perret et al. (This volume Perret et al., n.d.) develop a multi‐parameter mathematical model to explore the theoretical impact of cancer on predator–prey relationships, such as by affecting run speed, demonstrating complex impacts on ecological dynamics of populations, particularly for predators. Cancer can exert selective pressure on species, such as by conferring increased susceptibility to predation or reduced hunting efficiency, to promote the evolution of resistance strategies, compensatory changes in fertility, and feedback loops between predators and prey. There are many testable predictions from this work, including that loss of predators should increase cancer rates in their prey, and careful field work will be required to test these ideas.

While we know that cancers occur across metazoans, detecting and monitoring cancers in wild animal populations are fraught with difficulties. Hamede et al. (This volume Hamede et al., n.d.) discuss contagious oncogenic processes (from viruses to transmissible cancers) in the etiology of cancers in wild animals, and their importance in conservation efforts. A diverse and functional immune system at the population level may be important for limiting the spread of both virus‐induced and transmissible cancers, just as these viruses and cancers themselves often evolve mechanisms to subvert host defenses. Host animals with cancers can even alter life history traits, such by earlier reproduction, in an attempt to maintain fitness despite cancer pathogenesis (a mechanism of tolerance). Cancers are clearly agents of selection, which can lead to co‐existence of the cancers and the host over evolutionary time (such as dogs in response to canine transmissible venereal tumor—CTVT) or increased tolerance/resistance over a few generations (for Devils in response to devil facial tumor disease ‐ DFTD). The dramatic rise in DFTD in the last few decades has allowed documentation of the cascading albeit indirect impact of this tumor on other species and the overall ecosystem. The authors also highlight the impact that human activities have on cancer prevalence, including from habitat destruction, environmental carcinogenic contaminations, and climate change. They emphasize the need for improved strategies of surveillance, investigation, and mitigation. In all, the authors promote the critical need for a multidisciplinary approach to understand cancers in wildlife at multiple levels (overall ecology, population, individual, and tumor) and timescales (across millennia, over generations, within a lifetime) in order to protect our nonhuman brethren—often from ourselves.

Just as Darwin recognized the importance of artificial selection and domestication for understanding natural selection (Darwin, 1876), Thomas et al. (This volume Thomas et al., n.d.) describe how human‐driven selection for desired traits can lead to high cancer rates, and more intriguingly how compensatory tumor suppressive mechanisms can be selected for even when such processes might be “too expensive” for wild animals, given greater resource availability, reduced threats and often absent competition associated with domestication. Population bottlenecks, high homozygosity, and linkage disequilibrium with desired traits can also contribute to fixation of oncogenic traits in domesticated animals. We can learn a lot about cancer etiology from the products of thousands of years of animal domestication.

Like many birds, sea gulls have evolved long lifespans and presumably also similarly delayed cancer rates. Flight endows creatures with a potent mechanism of predator avoidance, and this lowered extrinsic mortality provided an advantage to a longer lifespan (providing further opportunities for reproduction). Meitern et al. (This volume Meitern et al., n.d.) examined gene expression in whole blood from young and old (>16 years) sea gulls, with a focus on genes involved in cancer. Interesting, the vast majority of changes from young to old involved downregulation of gene expression. In particular, they observed reduced expression of eight cancer‐related genes in old birds, and they speculate on how these changes could contribute to cancer risk. While determining how these expression changes might contribute to increased cancer risk in old age would require reverse genetic studies, which is not currently feasible for this species, these pioneering studies highlight the potential of comparative biology using wild animal populations to reveal new mechanisms controlling cancer susceptibility.

While we typically focus on the products of scientific research, studies of the research process are rare, and yet we can learn from research experiences how to improve future studies. Dujon et al. (This volume Dujon et al., n.d.) analyzed international collaborative networks for research on transmissible cancers. International and interdisciplinary research collaborations can allow researchers to tackle complex systems that would otherwise prove intractable. The authors used bibliometric and social network analyses to conduct a meta‐analysis of collaborative efforts directed at the three known transmissible cancers. They analyzed how these organized collaborations form and increase over time, providing information into what organizational forms for such collaborations are optimal for information sharing. In particular, while the small‐world type (dense) networks formed may facilitate information sharing, the small worldness values between institutions studying Devil transmissible tumors (DFTD) are lower relative to those studying the other two transmissible cancers, indicating that collaborations are not optimal for DFTD research. Their results suggest how efforts could be orchestrated, such as through rapid formation of multidisciplinary international teams with expertise in relevant areas (from ecology to immunology), to maximize the response to the emergence of a new transmissible cancer.

## 5 CONCLUDING THE CONCLUSIONS

Science should still be driven by the acquisition of new knowledge for knowledge's sake. When we understand fundamentals, whether at evolutionary, organismal, cellular, or molecular levels, we build a foundation upon which “useful” discoveries can be made. From this special issue of Evolutionary Applications, we have gained fascinating new insight into why we and other animals get cancer, how somatic evolution is either impeded, tolerated, or promoted within us in a context‐dependent fashion, how cellular dynamics within a cancer can dictate its trajectory (including its metastatic spread), and how cancers influence wild animal populations and overall ecology. But beyond the why and how, we have also gained a lot of useful information that should inform the development of improved methods of prevention and treatment for cancers. Studies are showing how we can manipulate cancer trajectories by playing cancer cells against each other and approaches to delay the evolution of resistance. Additionally, an appreciation of the critical role of both the tissue and tumor microenvironments in shaping cancer initiation and development should spur the development of methods to prevent and tame malignancies. While these approaches are not yet standard‐of‐care, there is confidence that with time and a substantially more investigation that evolutionary approaches to preventing and treating cancers will transform the oncology clinic and lead to great benefits for all of us (including nonhumans). To get there, evolutionary guided research and thought need to move beyond the fringes of cancer biology, to permeate textbooks, journals, classrooms, and laboratories.

## CONFLICT OF INTEREST

None declared.

## Data Availability

There are no data presented in this Conclusion for the Special Issue.
